# Focusing on the Environment to Improve Youth Participation: Experiences and Perspectives of Occupational Therapists

**DOI:** 10.3390/ijerph121013388

**Published:** 2015-10-23

**Authors:** Dana Anaby, Mary Law, Rachel Teplicky, Laura Turner

**Affiliations:** 1 School of Physical and Occupational Therapy, McGill University, 3654 Promenade Sir-William-Osler, Montreal, QC H3G 1Y5, Canada; 2 CanChild Centre for Childhood Disability Research, McMaster University, 1400 Main Street West, Hamilton, ON L8S 1C7, Canada; E-Mails: lawm@mcmaster.ca (M.L.); teplicr@mcmaster.ca (R.T.); 3 CBI Health Group, 3300 Bloor Street West, Toronto, ON M8X 2X2, Canada; E-Mail: lauraturner.ot@gmail.com

**Keywords:** participation, leisure activities, environment barriers, intervention, childhood disability, adolescence, occupational therapy

## Abstract

The environment plays a key role in supporting children’s participation and can serve as a focus of intervention. This study aimed to elicit the perceptions and experiences of occupational therapists who had applied the PREP approach—Pathways and Resources for Engagement and Participation. PREP is a novel 12-week intervention for youth with physical disabilities, aimed at improving participation in leisure community-based activities by modifying aspects of the environment. Using a qualitative post-intervention only design, 12 therapists took part in individual semi-structured interviews, in which the therapists reflected on their experience using PREP to enable participation. A thematic analysis was conducted. Four themes emerged from the data; two of which were informative in nature, describing elements of the PREP intervention that target multi-layered composition of the environment and use strategies that involve leveraging resources and problem solving. The two remaining themes were reflective in nature, illustrating a new take on the Occupational Therapy role and re-positioning the concept of participation in therapy practices. Results emphasize aspects of the environment that can serve as effective targets of intervention, guided by the PREP approach. Findings can broaden the scope and focus of occupational therapy practice by redefining views on participation and the environment.

## 1. Introduction

Participation of youth in activities at home, school, and in their community is one of the most important outcomes of rehabilitation interventions because it is fundamental to their health and development [1]. The participation of youth with physical disabilities; however, is significantly restricted in comparison to their typically-developing peers [2,3,4], and those discrepancies increase with age, making the transition to adulthood even more complex. One of the predominant reasons for this restriction lies within the environment [5,6]. Emerging therapy interventions for children with physical disabilities, suggest focusing change on the environment and/or the activity demands (*i.e.*, context-focused therapy) rather than directing change on the child’s abilities (*i.e.*, child-focused therapy) as a useful approach to improve function and participation [7].

Our research team has recently developed and tested a new environment-based approach called PREP (Pathways and Resources for Engagement and Participation) [8,9,10]. PREP is a client-centered strength-based approach that aims at improving youth participation in three community-based activities by solely modifying aspects of the environment. PREP is targeted for youth ages 12 to 18 with various functional issues. Within this environment-based intervention, the therapist, youth and family work together to identify environment-related barriers and/or facilitators (e.g., physical, social, institutional, activity demands) to participation in each of the three activities. Solution-based strategies to remove barriers are then proposed and implemented while building on youth/family strengths and supports. This process involves a coaching element [11,12], where the therapist engages and coaches both parents and youth on how to identify and apply effective strategies to improve the youth’s participation. A detailed protocol has been developed describing the five steps of the intervention: (1) make goals; (2) map out a plan; (3) make it happen; (4) measure the process and outcomes; and (5) move forward [9]. This protocol has been demonstrated to be effective in two different geographic locations. The effect of the intervention has been replicated across more than 40 leisure community-based activities/participation goals (e.g., playing baseball and hanging out with friends) that were set by youth with physical disabilities [8,10]. An analysis of this effect shows a significant improvement, *i.e.*, about two times as much, in self-rated levels of performance of the selected goals (mean = 4.5 on a 10-point scale, SD = 1.6) using the Canadian Occupational Performance Measure [13].

While improvement in youth participation has been established, it is also important to gain an in-depth understanding of the process, usefulness, and effectiveness of the intervention from the therapists’ perspective. Such knowledge can inform the development of knowledge translation strategies and training programs aimed at transferring the required skills embedded in this intervention to other therapists. The objective of this study; therefore, is to elicit/explore therapists’ perspectives on and experiences of implementing/delivering the PREP intervention.

## 2. Experimental Section

### 2.1. Procedure

A qualitative post-intervention only study design was employed. All occupational therapists (*n* = 12) who delivered the intervention provided informed consent to take part in an hour-long interview in a quiet location of their choice. Three interviewers independently conducted the interviews that took place no more than two to four weeks post-intervention. They followed a semi-structured interview guide consisting of five open-ended questions, as well as follow-up prompts in order to enhance optimal and focused responses (see Table 1). This interview guide was reviewed by two experts to reduce ambiguity and leading questions. Two of the three interviewers were independent of the study and all were rehabilitation healthcare professionals in pediatrics with similar levels of experience in interviewing and common understanding of the studied approach. The interview elicited the occupational therapists’ experiences and impressions of the intervention, including its format, process of identifying barriers to participation and of developing strategies to overcome these barriers, as well as their perceptions of the overall usefulness of the intervention. All interviews were recorded and Transcribed verbatim. Ethics approval was granted by the CER (Comité d’éthique de la recherché) of The Center for Interdisciplinary Research in Rehabilitation of Greater Montreal (CRIR-865-0713; October 2013). To ensure confidentiality, aliases were used for this report.

**Table 1:** Interview guide for therapists.

Questions	Prompts
Tell me about your overall experience and impression of the intervention.	(a) What do you think about the structure of the intervention? Prompts: duration, number of sessions, assessments (COPM scale), location of treatment, scheduling, procedures, data collection, completing forms. (b) What was the response of the adolescents and family to the intervention? Prompts: rapport, therapist-participant relationship, following the study protocol.
2. This intervention is focused on setting goals, identifying barriers to participation and implementing strategies to overcome them: can you tell me more about this process?	(a) Describe the process of setting goals (using the assessments, discussion with youth/parents, feasibility of goals, solutions). (b) What were the most common barriers related to the environment that the participants identified? (c) What were the strategies that you used in order to remove these barriers? (d) Can you give me examples of moments when you thought you had been effective in removing barriers? What was that like? (e) Can you give me examples of moments when you thought you had been less effective in removing barriers? What was that like?
3. What additional aspects of the intervention do you think were effective in enhancing each youth’s participation? What aspects do you think were ineffective?	
4. Can you think of circumstances (unrelated to the intervention) that may influence the adolescent’s participation?	(a) Prompts: family life events, season, specific goals.
5. Can you suggest ways in which the intervention can be improved in the future?	(a) What would you do the same? (b) What would you do differently?

### 2.2. Data Analysis

The content of each interview was analyzed using rigor thematic analysis techniques, as described by Braun and Clarke [14] providing support to the trustworthiness of the findings. An initial coding scheme was created based on the first four interviews, where each interview transcript was read and discussed by two researchers. The researchers then identified the initial codes that reflected meaning within each transcript separately. The same initial codes were then organized into meaningful groups. This process allowed the data to be categorized into overarching themes. The final eight interviews were coded by drawing upon the initial scheme and on meaningful groups and themes. Throughout this process, the coding scheme and emerging themes were discussed during ongoing meetings with another researcher. Finally, themes have been checked against each other and back to the original data set and this process was supported by a team of researchers and expert input. QSR International’s NVivo-10 qualitative analysis software was used for the coding process and the data organization.

## 3. Results

### 3.1. Sample Characteristics

Twelve female occupational therapists (OTs) who delivered the PREP intervention to youth with physical disabilities, such as cerebral palsy and spina bifida, ages 12 to 17 participated in the study. The majority (*n* = 10) had a Master’s degree and their years of clinical experience ranged from 6 months to 20 years (mean = 5.9). More than half of the therapists worked full-time in settings including rehabilitation centers (*n* = 8) and the others worked in schools (*n* = 1), community agencies (*n* = 1), hospitals (*n* = 1) and research centers (*n* = 1). Of the sample of 12 OTs, 10 had taken a clinical educational course within the past two years, highlighting their commitment to continuing education and enhancing their practice.

### 3.2. Main Findings

Four overarching themes have emerged from the data. Two of the themes are informative in nature, describing elements of the intervention: multi-layered composition of the environment and leveraging resources and problem solving. The remaining themes are reflective in nature, illustrating a new take on the OT role, as well as re-positioning the concept of participation in therapy practices. Specifically, emerging insight surrounding the concept of participation and its benefits were also identified. Examples included going beyond the clinical setting; the importance of providing intervention in the actual place where the youth/family is participating, including the home and community; participation efforts are positive and contribute to the sustainability of increased participation.

### 3.3. Informative Themes

#### 3.3.1. Multi-Layered Composition of the Environment

Therapists encountered a vast array of environmental barriers to participation that varied across families. Different facets of the environment, (*i.e.*, physical, attitudinal, social and institutional) served as barriers to participation. Examples include: availability of programs (adapted/eligible for this age range or certain diagnosis), adapted/required equipment, transportation, the physical/built environment (physical accessibility), geographic location and financial barriers. Temporal elements of the environment (*i.e.*, scheduling, time, seasons/weather) were also difficult to negotiate and required a great deal of flexibility for all parties. The attitudinal factor seemed complex and often challenging; it involved parents’ perspectives towards community participation (*i.e.*, anxiety, hesitation) and towards independence and autonomy. Some parents were reluctant about their child going out on their own and participating in the environment, particularly when engaging in unstructured activities, such as “going to the mall with friends”. Another attitudinal barrier was related to the perceptions of others and willingness of organizations and instructors from community-based programs to adapt their programs to enable participation of youth with disabilities.

To illustrate, attitudes of community organizations were observed when trying to work on the participation goal of a 17-year-old youth who uses a wheelchair for mobility. The youth’s goal was to “volunteer with kids”, and the therapist stated:

“The main barrier (of this goal) was finding the right place that could take (the participant) on as a volunteer without adding more responsibility to the staff”.

*(Gail)*

Another therapist described the eligibility of a piano program offered by a non-profit organization as an example of an attitudinal support:

“That (organization) was for adults only, or for people who don’t go to school at all. He [the participant] already goes to school full time and he’s a youth, so it didn’t, at first, make him eligible. I ended up speaking to the director of the non-profit as well as the registrar and they sort of understood the situation and they were able to accommodate him, which was really good”.

*(Lucia)*

Social aspects of the environment, including family support and friendship, served as an important barrier and/or support to participation. The role of the family was identified quite often by illustrating a blend of barriers and supports, and the family as a unit (at times involving siblings and cousins) was very intertwined in the process of the intervention. Therapists felt that parents had to be on board with the project just as much as youth, as they would facilitate some of the more logistical aspects, like transportation or finances. The parents’ ability and willingness to assist, determined whether this element was a support or barrier to participation. Parents’ familiarity with participation was an important facilitator, yet it varied. Different families also had varying knowledge and understanding of the available community resources; some families were very involved in community activities already, whereas other families had never branched out to try community activities. Esther (OT) illustrates:

“I think (the mom) felt isolated she told me she had felt kind of isolated because they are not really part of a big community. I just don’t think they knew where to start, really. So I think they were appreciative just knowing that I was here”.

Another therapist, Deborah, comments about another family:

“I think that it opened their eyes to what is possible and what they could do with their child and what they could do with their family. I think it was very beneficial for the family in that way”.

At times, parent and youth motivation and ideas differed, reflecting their dynamic relationships. For example, one youth refused to do any goals suggested by their parent, yet parents of other youth provided guidance in the goal-setting process.

#### 3.3.2. Leveraging Resources and Problem-Solving

Therapists developed a range of solution-based strategies to overcome barriers in the environment and strengthen facilitators by employing an individualized, client-centred approach to meet each family’s unique needs. Some strategies were described as “simple solutions” and resulted in a quick change in outcomes, e.g., searching for and exchanging information with the family about a certain community program. This was illustrated by Lucia:

“It was really to find the resources and to connect them with the child as well as the family that was pretty much the most significant type of intervention that I had to do”.

Seeking out resources including, for example, programs in the community, financial assistance or low-cost programs, as well as shadows/volunteer-based services, were all strategies used to facilitate participation. In most cases, however, strategies were more complex in nature and required a unique and creative approach of problem solving, leveraging existing resources and building on supports in order to facilitate participation. These strategies involved liaising and working together with different stakeholders or “community partners” such as colleagues, other therapists (physical therapists, social workers), physical rehabilitation technicians, graduate/high school students, teachers, volunteers, health care vendors, program instructors, directors and other service providers. At times, creating new resources to support participation was reported, such as developing a group of volunteers in a local high school to facilitate the goal of “enjoying music within a social group/setting” and a team of occupational therapist assistants to facilitate the goal of “participating in amusement rides” and problem solve through how to get the youth on and off the rides. This collaborative process involved an educative element in which therapists guided stakeholders about the best way to provide accessible services. As Alexandra indicated when working with a 17 year old girl who wanted to ride a bike with her family and friends in her neighborhood:

“So I spent a lot of my time trying to find someone who would be suitable, and I found a Master’s student who is studying Adaptive Physical Activity at (name of a University) who is willing to do it (work with the youth) on a volunteer basis. And she came and met the client and I went over some things she could do to practice it”.

Problem-solving strategies were also used to address youth-related factors that presented themselves as barriers and/or facilitators to participation, such as attributes/traits, preferences and self-perceived abilities and skills. Examples included aspects of the personality (e.g., shyness, persistent, resilience, motivation); physical capability doubts (e.g., lack of self-confidence in abilities and skills was addressed by trying the yoga Wii game at home prior of taking class in the community); as well as cognitive and communication skills (e.g., bilingual player specifically assisted participants during boccia game). To address family-related barriers, such as parents’ anxiety about youth hanging out in the mall with friends, a structured solution-based plan was proposed and involved technology use to keep in touch, having a plan around toileting, trying a two hours mall visit first, going over the bus route using iPad and suggesting strategies to use when things do not go to plan. Participation efforts were perceived as positive and, in return, influenced aspects of the youth; they contributed to enjoyment, to a sense of achievement, pride and motivation, once the barrier was removed.

### 3.4. Reflective Themes

#### 3.4.1. A New Take on the OT Role

The therapists’ experience led to a new, refreshing take on the OT role, which was perceived as a unique method of practice and a different type of service by both therapists and parents. To illustrate:

“I thought it was really nice experience. Very different than what I do on a daily basis, which is great, because it shows a different side of OT and I really like the idea of finding these kids and helping them participate more in the community. It’s great and they should have that opportunity to do so and to find more resources”.

*(Gail)*

The occupational therapist was specifically described as a key facilitator, an advocator, an educator and a team collaborator; one that links participants to the community and operates in a new fashion outside of the clinic. Through coaching of youth, parents, volunteers and service providers, therapists allowed for capacity building and promoted a sense of empowerment. To illustrate, in some cases, families “took charge” or “took the lead” and were able to find activities and/or resources on their own, based on strategies provided by the therapists. Therapists also provided support and reinforcement and were often described as a “mentor”, one whose presence in the setting where activity occurs was encouraging. Overall, this model of practice was perceived as different and unique; “I didn’t do a lot of hands-on intervention” (Melina).

Some therapists expressed preconceived ideas about occupational therapy practice and services, indicating that this intervention “is not therapy”, *per se*. Their previous experience reflected at times impairment-based and/or condition-based types of intervention. As Gabrielle mentioned:

“We are more facilitators, we are helping them finding resources, establishing links, getting confidence in that networking. But we are not doing therapy. The intervention is really a mentorship facilitating process.”

Others felt that this experience re-affirmed their own beliefs about practice and appreciated the focus of this therapy intervention, *i.e.*, modifying aspects of the environment rather than improving skills or changing other aspects of the youth.

“I enjoy that type of rehab much more. I’m not a very therapy-room, you know basket of toys on the floor-type. That’s not my style. I’m much more into adapting the environment and adapting equipment” .

*(Alexandra)*

Overall, this process led to reframing outlooks about participation, as described in the following theme.

#### 3.4.2. Re-Positioning the Concept of Participation

Delivering the PREP approach led to an evolvement of insights about the concept of participation and its benefits. Therapists discussed the importance and relevance of participation to this specific age group (adolescents), in particular engaging in leisure activities. The emerging awareness of participation as a focus of intervention is illustrated by Kalini’s observation:

“I think that it’s a good reminder of the importance of participation and what the barriers are, because it’s something that I think is important. I think one thing this study has shown me has taught me a lot so far in the last two sessions with the clients is how much the family situations – the family income, where they live, their ability to be involved, the transportation really has big impact—and I think those barriers or facilitators, depending on what they are, have a huge impact on child’s ability to participate. But I think that we can’t deny the effect the family has on participation”.

Therapists also acknowledged the unique benefit of “going beyond the clinical setting” and providing services in multiple settings/locations within the youth’s own natural environment (e.g., community, school, home). Such an approach was perceived as practical and effective and believed to facilitate sustainability, as described by Esther:

“I think being integrated into the community, being part of their home and being onsite really made it all work. Being able to do the walk and ride the bike riding route in the neighbourhood and to know their family.”

As well as by Vera:

“I think it is more, kind of, personal or appropriate in your own environment right. Because for it to actually work out in the long term you want it to be, you know, convenient and easily accessible. So for this particular family, they live in (suburb city). So finding resources in that city was very important.”

When reflecting on the meaning of participation, a few therapists were questioning their own current views of the concept. This involved, for instance, the way they perceived successful participation, recognizing that there are many ways to be and feel engaged in an activity and that the experience of participation is not always dependent on skill acquisition. What youth can do in a supportive and welcoming environment, as opposed to what they cannot do, is what matters most and can be very empowering and motivating. Melina illustrated this:

“We (therapists) really need to just put away our ideas of what success actually is and go with the perception of the client, and if they had fun and feel it was successful, then I think we’ve done a good job”.

## 4. Discussions

This study sought to explore the perceptions and experiences of therapists who had implemented the PREP approach, an environment-based intervention for youth with physical disabilities aimed at improving their participation in the community. Various facets of the environment were acknowledged as barriers to participation and as a target of intervention. Notably, environmental factors were not limited to physical accessibility or the built environment—often considered as a predominant element in practice—and included other aspects such as attitudinal, social and institutional factors. These findings coincide with models in occupational therapy, such as the Occupational Therapy Practice Framework [15] and the Person-Environment-Occupation Model [15,16], as well as previous research exploring the impact of the environment on youth participation [5]. Consequently, they can further increase awareness of the broader scope of the environment that can be addressed in clinical practice. In addition, therapists recognized the benefits of interventions that are provided within their client’s own environment. This finding is well aligned with current therapy approaches for children and youth with cerebral palsy [17]—those that encourage family-centered and ecological-based approaches to interventions. Such approaches have been proven effective in promoting child function and participation [18] and, in combination with the therapists’ perspective, can further enhance evidence-based practice.

The evolving perceptions about the PREP approach and the OTs’ practices were also important findings. Therapists’ preconceived ideas about the OT role and OT services emerged, at times challenging the therapeutic nature of the PREP intervention, despite its positive effect on participation outcomes. Previous research among clinicians who had also implemented another environment-based approach, *i.e.*, context therapy, revealed a similar perspective; clinicians did not perceive it as “true” therapy if “hands-on” treatment was not provided [7]. Although the PREP approach fits with models of OT theory and intervention, re-directing therapists’ attention towards a focus on the environment and increasing awareness and added value of this approach is still required.

In-line with current approaches in occupational therapy [15,19,20], therapists used a range of clinical skills and intervention types in order to remove barriers in the environment. These skills go beyond “traditional” or “typical” therapy and included, for example, advocating, coaching/training, educating and collaborating, among others. This treatment approach has its merit, particularly the coaching aspect; it can foster youth and parent knowledge, problem-solving and self-advocacy skills, and can help them develop strategies to overcome barriers autonomously and, consequently, facilitate sustainability and maintain levels of participation. Building supportive attitudes of therapists about the value and relevance of such skills and methods of practice that are inherent/deeply rooted in occupational therapy services is therefore warranted. This can be achieved through effective knowledge translation initiatives aimed at training therapists about useful strategies to improve youth participation and, consequently, promoting the uptake of the PREP approach into practice. Initial knowledge translation strategies include activities to raise occupational therapists’ awareness and knowledge about the approach, including sharing educational materials through a website, conferences and/or workshops. These strategies should be followed by more interactive approaches to promote changes in practice, such as case based tutorials/learning sessions to facilitate team discussions and problem-solving.

### Limitations and Further Directions

All therapists delivered this intervention as part of a study investigating the impact of PREP on youth participation. Examining the implementation of this approach in actual clinical routine is warranted and may reveal additional perspectives and experiences, such as those related to the organization’s culture and funding structure, among other realities of clinical practice. While clinicians’ input is key in the process of facilitating the adoption of a new intervention into the clinical setting, it is also important to incorporate and triangulate youth and parents’ perspectives. Such a comprehensive examination can further contribute to our understating of this model of practice and its implications. Finally, while a number of strategies were used to support trustworthiness in this qualitative study, member-checking was not used as a method to verify interpretation of the findings due to time constraints. This may have had an impact on the findings.

## 5. Conclusions

Our findings emphasize the broad aspects of the environment that can serve as barriers and/or facilitators to youth participation. These barriers can be targeted and modified effectively by using the PREP principles. The emerging perspectives of the OT role and the concept of participation can potentially expand the scope and vision of the occupational therapy profession.
